# Acute Pancreatitis Secondary to Pembrolizumab-Induced Hypertriglyceridemia

**DOI:** 10.7759/cureus.38315

**Published:** 2023-04-29

**Authors:** Ammar Ashfaq, Nishanth Thalambedu, Muhammad Umair Atiq

**Affiliations:** 1 Internal Medicine, Jefferson Abington Hospital, Abington, USA; 2 Internal Medicine, University of Arkansas for Medical Sciences, Little Rock, USA; 3 Internal Medicine, Thomas Jefferson University Hospital - Jefferson Health, Philadelphia, USA

**Keywords:** hypertriglyceridemia-induced acute pancreatitis, hypertriglyceridemia, pembrolizumab, cancer immunotherapy, acute pancreatitis

## Abstract

Pembrolizumab is a humanized monoclonal antibody targeted against programmed cell death protein 1 (PD-1) receptor of lymphocytes. It is used alone or in combination with many chemotherapy regimens for a wide variety of cancers. It has been reported to cause various side effects including endocrinopathies, colitis, rash, and pneumonitis. Hypertriglyceridemia (HTG) has been recently added to its side effect profile with a possible pathogenic mechanism involving autoantibodies against glycosylphosphatidylinositol-anchored high-density lipoprotein binding protein 1 (GP1HBP1). We are presenting a case of acute pancreatitis secondary to HTG in a cervical cancer patient. HTG was successfully treated with insulin infusion. As the patient’s symptoms improved, she was started on the diet. She was discharged on statin and fibrate therapy. We are reporting this case to increase awareness of this rare side effect, inpatient management, and outpatient screening while on immunotherapy.

## Introduction

Immune checkpoint inhibitors have been a marvelous addition to anticancer therapy. They enhance the antitumor and cytotoxic activity of T lymphocytes. Two types of immune checkpoint inhibitors have been developed; one targets cytotoxic T lymphocyte antigen 4 (CTLA-4) and others act on programmed cell death 1 receptor (PD-1). Pembrolizumab, a widely used immunotherapy antitumor drug, is a humanized monoclonal antibody against the PD-1 receptor. Although, pembrolizumab is tolerated well with fewer immune-related adverse events (irAE), including endocrinopathies, colitis, rash, hepatitis, and pneumonitis [1]. However, its side effect profile continues to grow whereas hypertriglyceridemia (HTG) has been recently reported as a potential side effect [2,3]. In this study, we present a case of a cervical cancer patient on pembrolizumab who went on to develop HTG culminating in acute pancreatitis (AP).

## Case presentation

A 43-year-old Hispanic female with a body mass index of 30.6 kg/m^2^ presented to the emergency department for progressive epigastric pain of 48 hours. The patient's past medical history was significant for hypertension, hypothyroidism, and stage IVB cervical cancer status post-treatment with cisplatin and radiation for six weeks. The patient was recently started on carboplatin, paclitaxel, bevacizumab, and pembrolizumab almost two weeks before the presentation. Home medications were nifedipine 60 mg per day, hydrochlorothiazide 25 mg per day, and levothyroxine 137 mg daily. Family history was significant for hypertension.

**Figure 1:**
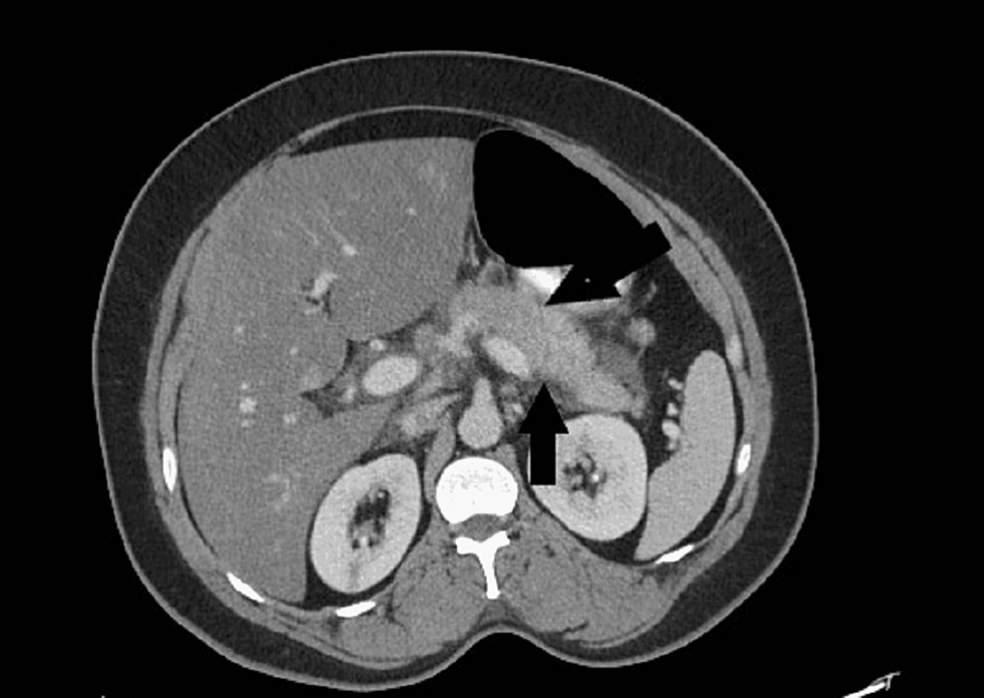
CT scan abdomen showing irregular pancreatic contour and diffuse peri-pancreatic swelling.

**Figure 2:**
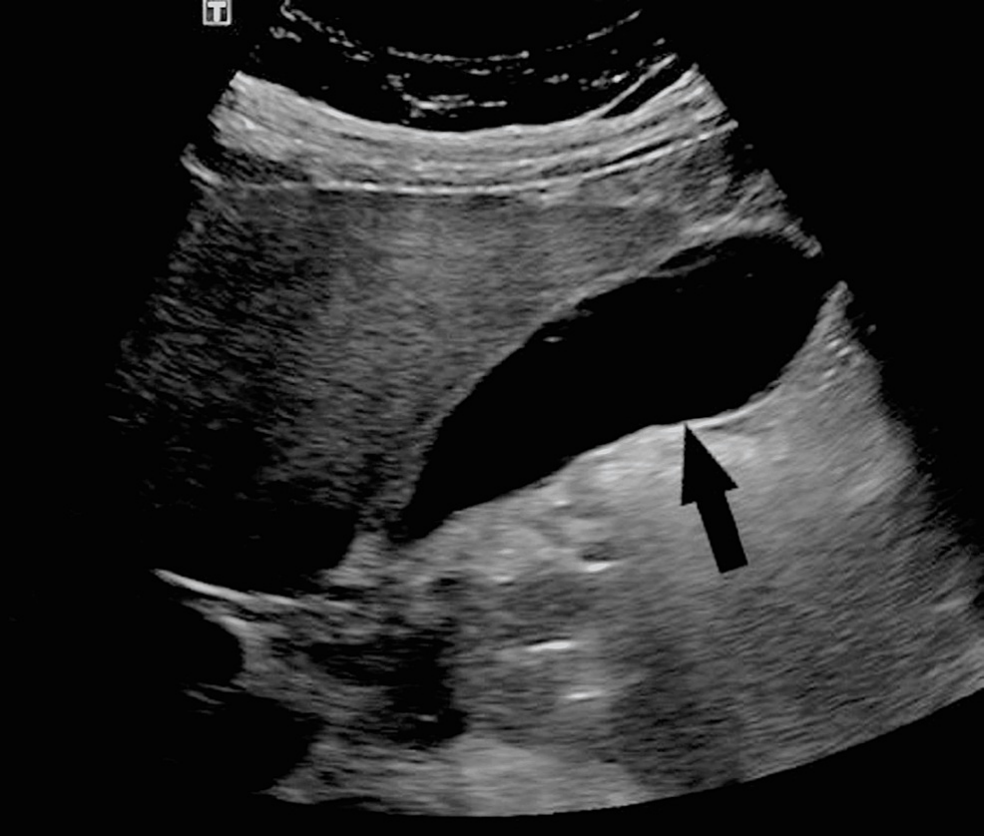
Ultrasound abdomen shows no gallstones.

The patient's abdominal pain was associated with nausea and radiating to the back. The patient also complained of decreased tolerance to eating and drinking liquids. The patient denied any history of alcohol abuse or drug intoxication. Vital signs in the emergency department were as follows - blood pressure was 165/94 mmHg, respiratory rate was 17 breaths/minute, heart rate was 98 beats/minute, temperature was 99°F, and oxygen saturation was 95% on room air. A blood workup was obtained, and a computed tomography (CT) scan of the abdomen and pelvis was done which revealed diffuse peri-pancreatic edema with a focal fluid collection representing acute pancreatitis (Figure 1). Edema is tracked along the second and third portions of the duodenum and into small bowel mesentery. She also had an ultrasound abdomen which was unremarkable (Figure 2). The patient was made nothing by mouth (NPO) and was started on intravenous fluids along with pain medicines. The patient's blood workup on presentation has been shown in Table 1.

**Table 1:** Laboratory findings at presentation.

Laboratory test	Value	Reference range
Lactate dehydrogenase (IU/L)	230	125-240
Lipase (U/L)	2387	0-95
Amylase (U/L)	282	0-130
Total bilirubin (mg/dL)	0.4	0.3-1.2
Aspartate aminotransferase (U/L)	19	0-35
Alanine aminotransferase (U/L)	13	0-35
Calcium (mg/dL)	7.3	8.5-10.3
Total protein (g/dL)	6.4	3.2-4.9
Albumin (g/dL)	3.2	3.2-4.9
Blood urea nitrogen (mg/dL)	11	8-20
Creatinine (mg/dL)	0.56	0.7-1.3
Sodium (mmol/L)	132	135-146
Potassium (mmol/L)	3.9	3.5-5.0
Chloride (mmol/L)	101	89-109
Bicarbonate (mmol/L)	20	21-30
Anion gap (mmol/L)	11	6-14
Glycated hemoglobin (%)	6.9	5.7-6.4
White blood cell count (k/uL)	7.2	4000-10,000
Hemoglobin (g/dL)	11.3	12.0-16.0
Platelets (k/uL)	151,000	150,000-350,000
International normalized ratio (INR)	1.10	0.82-1.13
Partial thromboplastin time (sec)	25	25-37
Thyroid stimulation hormone (uIU/L)	85	0.30-5.0
Free triiodothyronine (pg/mL)	1.4	2.0-4.4
Free thyroxine (ng/dL)	0.9	0.7-1.7
High-density lipoprotein (mg/dL)	26	>60
Cholesterol (mg/dL)	435	150-250
Low-density lipoprotein (mg/dL)	29	<100
Triglyceride (mg/dL)	2043	70-290
Serum alcohol level (mg/dL)	<10	≤9
Serum acetaminophen level (mcg/mL)	<5	≤25.0

**Figure 3:**
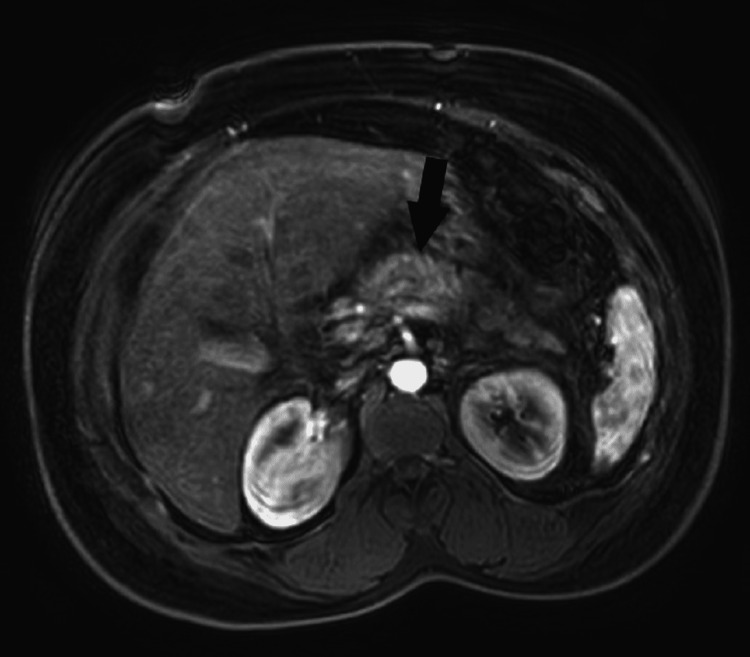
MRI abdomen with MRCP showing irregular contour of pancreas and swelling. MRCP: magnetic resonance imaging with cholangiopancreatography

The patient also had magnetic resonance imaging with cholangiopancreatography (MRCP) which showed similar findings as the CT scan with normal pancreatic and bile duct (Figure 3). The patient was evaluated by gastroenterology and the decision was made to start her insulin drip for hypertriglyceridemia-induced pancreatitis. The trend of triglyceride (TG) level and lipase on insulin infusion is shown in Table 2. She was transferred to the medical intensive care unit for closer monitoring. The patient's triglyceride levels were appropriately decreased below 500 mg/dL and then the patient was started on subcutaneous insulin glargine. The patient was downgraded to the general medical floor. The patient started to tolerate clear fluids with carbohydrates and the diet was slowly advanced to a low-fat diet. The patient was discharged on atorvastatin and fenofibrate. She followed up with her primary care provider and oncologist in one week. She also had a repeat blood workup done and TG and lipase levels were normal as shown in Table 2.

**Table 2:** Triglyceride and lipase levels during and after hospitalization.

Day of admission	Triglyceride level (mg/dL)	Lipase level (U/L)
01	2043	2387
02	1068	624
03	944	-
03	785	271
04	663	241
05	598	-
06	572	-
07	400	-
08	351	-
09	335	-
10	313	-
11	295	-
02 weeks after discharge	235	22

## Discussion

Pembrolizumab-associated hypertriglyceridemia is rarely reported. The exact mechanism of pembrolizumab-causing hypertriglyceridemia is unclear but the possible hypothesis might be due to the activation of autoantibodies against glycosylphosphatidylinositol-anchored high-density lipoprotein binding protein 1 (GP1HBP1) [2,3]. It is a protein molecule located in the capillary endothelial cells that help in the transport of the enzyme lipoprotein lipase (LPL) produced from adipocyte to the vascular lumen where it breaks down triglycerides into free fatty acids. Relative deficiency of GP1HBP1 from pembrolizumab thereby prevents the movement of LPL into the intravascular compartment leading to hypertriglyceridemia [2].

Pembrolizumab-associated HTG-causing acute pancreatitis is one of the serious albeit rare adverse effects. Although other immune-related metabolic disturbances have been reported more commonly from immunotherapy, disorders involving lipid metabolism were sparsely reported [4]. Given the widespread use of pembrolizumab in the treatment of various cancers in the last decade, an insight into its rare adverse effects is highly warranted.

Not all hypertriglyceridemia leads to acute pancreatitis (AP) and its pathophysiology is unclear. A possible mechanism might be due to the pancreatic ischemia caused by hyper-viscosity from the triglyceride-rich lipid particles in the circulation, chylomicrons, the largest of the lipoproteins. The pancreatic ischemia ultimately activates trypsinogen leading to AP [5]. Like other causes of acute pancreatitis, hypertriglyceridemia pancreatitis (HTGP) presents with acute abdominal pain, elevated serum pancreatic enzyme levels, and typical findings on imaging which were noted in our patient as well [6].

Early phases of AP irrespective of the cause were associated with mildly elevated levels of triglycerides and thereby leading to confusion in diagnosing HTGP. Although there is no clear cutoff for triglyceride level for diagnosis of HTGP, serum triglyceride level >1000 mg/dL was thought to trigger AP due to high triglycerides. Search for other causes of AP should be done, especially in subjects with mild-moderate elevations of triglyceride levels <1000 mg/dL [5]. Even though our patient was exposed to other drugs, however, the chance of causing HTGP because of them was insubstantial. Our patient's TG levels were noted to be 2043 mg/dL with classical clinical presentation of AP, which was consistent with HTGP.

Treatment of HTGP can be divided into two phases, general and specific. General treatment includes bowel rest, intravenous (IV) hydration, and IV analgesics for AP of any etiology [7]. Specific treatment includes treating the actual cause of AP, which was high triglycerides in our case. Although many treatments are known to reduce high TG levels, the most readily available with minimal expense is insulin infusion. Insulin activates LPL thereby fastening the process of high TG clearance from the intravascular compartment, removing the continuous trigger for AP [8]. Low molecular heparin use for severe acute pancreatitis has also been supported in literature but widespread use is limited due to the increased risk of bleeding complications [8,9]. Apheresis is another expensive and less readily available procedure to rapidly remove the high TG from the serum [10]. The use of more advanced antisense apo-B and apo-CIII inhibitors is currently limited to treating high TG levels in familial hyperlipidemic patients [8]. Once the TG levels were reduced to near normal levels they can be maintained by treatment with oral antilipidemic drugs like fibrates which acts by reducing TG synthesis in the liver and increasing LPL [7,11]. Our patient was initially treated in the intensive care unit with insulin infusion for rapid clearance of high TG and was switched to oral atorvastatin and fenofibrate on discharge. Her TG levels a few months later were within the normal limits.

## Conclusions

Although hypertriglyceridemia-related acute pancreatitis is well-established, pembrolizumab-associated HTG-causing pancreatitis has not been reported. Physicians should be made aware of and consider this rare drug side effect after ruling out the other most common cause of AP. Further studies and animal models are needed to study the exact pathophysiology and relation of this drug with hypertriglyceridemia. Our case also highlights that pembrolizumab-associated HTG-causing pancreatitis can be successfully managed with insulin therapy to lower serum TG levels. Frequent monitoring of triglyceride and lipase in patients receiving pembrolizumab needs to be investigated further for future monitoring of this rare side effect.
